# Patient Blood Management in Cardiovascular Surgery

**DOI:** 10.21470/1678-9741-2024-0994

**Published:** 2024-10-18

**Authors:** Isabel Cristina Céspedes, Maria Stella Figueiredo, Antonio Alceu dos Santos, Nelson Americo Hossne Junior

**Affiliations:** 1 Department of Morphology and Genetics, Discipline of Genetics, Escola Paulista de Medicina, Universidade Federal de São Paulo (UNIFESP), São Paulo, São Paulo, Brazil; 2 Department of Clinical and Experimental Oncology, Discipline of Hematology and Hemotherapy, Escola Paulista de Medicina, Universidade Federal de São Paulo (UNIFESP), São Paulo, São Paulo, Brazil; 3 Department of Clinical and Experimental Oncology, Postgraduate Program in Medicine - Hematology and Oncology, Escola Paulista de Medicina, Universidade Federal de São Paulo (UNIFESP), São Paulo, São Paulo, Brazil; 4 Department of Surgery, Discipline of Cardiovascular Surgery, Escola Paulista de Medicina, Universidade Federal de São Paulo (UNIFESP), São Paulo, São Paulo, Brazil

**Fig. 1:**
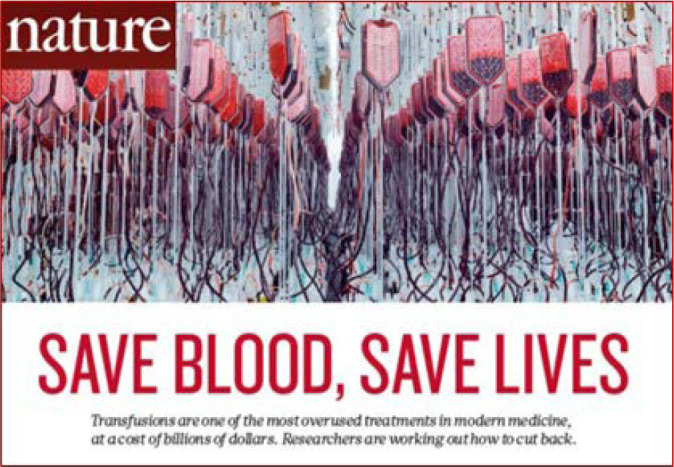
The article “Evidence-based medicine: Save blood, save lives” was published in the journal Nature in 2015^[1]^.

Blood transfusion is one of the most common therapeutic practices in medicine, becoming widely popular during the great world wars. Nevertheless, it has never undergone the necessary testing phases to ensure its efficacy and safety. Current evidence-based medicine concludes that this practice remains settled in medical practice without proper critical scrutiny of the subject. Current researchers have shown that the immunomodulatory and inflammatory effects of blood transfusions are significantly associated with worse clinical and surgical outcomes. A Nature editorial (2015)^[1]^ (Figure 1) listed the historical facts that have made blood transfusions so popular among physicians. However, the same editorial highlights that current scientific evidence demonstrates that allogeneic blood transfusions expose patient safety at risk by increasing hospital infection rates, length of stay, morbidity, and mortality, regardless of the patient's condition or comorbidities. The article also emphasizes the high cost of transfusions and the burden on healthcare systems.

In fact, important observational or randomized and meta-analysis studies have shown that, after excluding confounding factors (ethnicity; age; sex; comorbidities; elective, urgent, or emergency surgery; surgical team; etc.) and employing robust statistical analyses, allogeneic blood transfusions represent an independent risk factor for infection rate (21%), length of hospital stay (15%), and mortality (28%)^[2]^.

Observational studies have shown that transfusion of allogeneic red blood cell concentrates was associated with an increased risk of cardiac complications (arrhythmias, atrial fibrillation, myocardial infarction), renal failure, prolonged ventilatory support, stroke, mediastinitis, and serious infections, among others^[3,4]^.

These worse outcomes may be explained by underlying molecular events that have not yet been clinically identified. Studies initiated by our group to analyze the epigenetic effects of blood transfusions aim to identify some of these molecular events. It has already been observed that one of the main deleterious effects of blood transfusions on the recipient's immune system is the so-called transfusion-related immunomodulation, similar to what takes place in organ and tissue transplants, due to the high load of antigens injected into the recipient's circulation. This leads to a reduction in the number of circulating lymphocytes in the recipient, functional changes in T helper cells, and activation of immune cells^[5]^.

Blood transfusions also represent high costs for health systems. Even accounting for the low costs of the blood donation act per se, there are numerous production costs, paid per unit of blood bag by the public health system in Brazil (Sistema Único de Saúde - SUS), in addition to the costs of its infusion in a hospital environment. Additionally, the longer hospitalization time resulting from the transfusion of allogeneic blood leads to an increased healthcare burden. In 2023, the Joint Commission published a study reviewing the transfusion practices of American hospitals and demonstrated that 86.48% of transfusions were inappropriate or unnecessary at a cost of millions of dollars^[6]^. This is alarming from a patient safety point of view and public health management. Moreover, considering an ongoing aging population, it will be necessary to learn how to act without blood components due to the inevitable lack of active donors.

In view of this and other evidence, a paradigm shift has been discussed concerning allogeneic transfusion practice, strengthening the use of therapeutic options for blood transfusions, consisting of the Patient Blood Management (PBM) program. In general, this program is based on three pillars:

**1^st^ pillar (preoperative focus)**: mainly involves optimized treatment of anemias and coagulopathies and preparing the patient for surgical procedures.
**2^nd^ pillar (intraoperative focus)**: involves preserving the patient's blood, minimizing blood loss, and optimizing coagulation status through systemic and topical hemostatic drugs and products, blood cell recovery machines, and/or acute normovolemic hemodilution.
**3^rd^ pillar (postoperative focus)**: mainly involves the concept of anemia tolerance, oxygen support to optimize its supply, sedation to reduce oxygen demand, and reducing the frequency and volume of phlebotomies (such as fewer blood samples for laboratory exams), among other strategies.

All these strategies have already been published in the Brazilian Journal of Cardiovascular Surgery by Santos et al.^[7]^ in 2014. In Brazil, most of the tools, devices, and pharmacological strategies needed for PBM are part of the RENAME (Relação Nacional de Medicamentos or National List of Medicines) and RENEM (Relação de Equipamentos e Materiais or List of Equipment and Materials) lists, with low implementation costs.

**Fig. 2:**
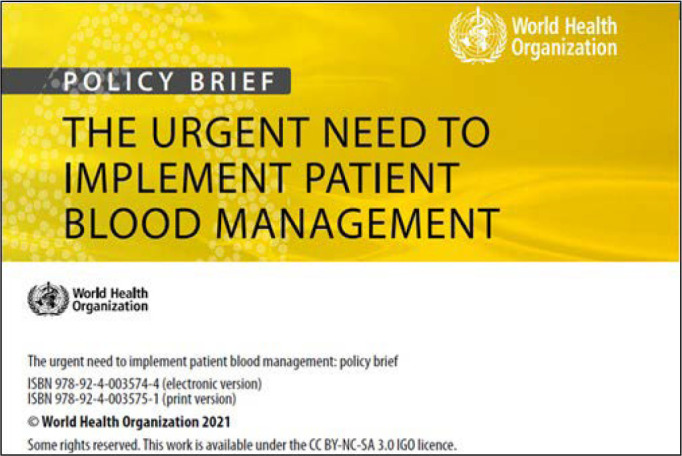
Document published by the World Health Organization in 2021, with the urgent call for the implementation of patient blood management policies, based on 229 scientific evidence in favor of this policy^[8]^.

A successful example of the PBM implementation was observed in Western Australia, with a reduction in the use of blood products (41% red blood cell, 47% plasma, and 27% platelets), resulting in 28% reduction in mortality rate, 31% reduction in acute myocardial infarction/stroke rate, 15% reduction in hospital length of stay, and 21% reduction in the number of infections, as well as savings of US$100 million in direct and indirect costs^[8]^. In Canada, the implementation of the PBM in 25 hospitals in the greater Toronto area has saved around 50 million Canadian dollars a year, over 20 years^[9]^. In 2021, the World Health Organization (or WHO) declared that it was urgent to implement PBM in hospitals (Figure 2) due to greater patient safety, savings in public health resources, a shortage of both blood components in blood banks and the number of blood donors, and the probable new pandemics^[8]^.

It is important to point out that the Enhanced Recovery After Surgery (or ERAS) principles already included PBM as a necessary strategy for best practice in cardiac surgery^[10]^.

A major setback in implementing PBM is the lack of adequate training in Transfusion Medicine, which is often underrated in medical curricula in Brazil or worldwide. A 2019 Brazilian study showed that 73% of Brazilian medical residents did not receive adequate training in Transfusion Medicine during their undergraduate course, and 93% would like to have received it. This is also the case in medical schools worldwide^[11,12]^. In fact, it can be seen in hospitals and medical and nursing schools that almost all these professionals don't even know about PBM.

In this sense, since 2018, the Group PBM-HU-UNIFESP (https://pbm.unifesp.br/) has been working on various fronts in favor of PBM:

**a) teaching**: created the elective discipline of Transfusion Medicine, focusing on PBM, in the Medical Graduation Course at the Escola Paulista de Medicina/Universidade Federal de São Paulo (UNIFESP), a pioneering initiative.
**b) training**: a compulsory PBM course for all medical residents, under the approval of COREME (Comissão de Residência Médica or Medical Residency Commission) of the Escola Paulista de Medicina/UNIFESP, which has also trained professionals from other teaching and assistance entities; another pioneering initiative.
**c) research**: line of research in Advanced Studies in Patient Blood Management - PBM by the postgraduate program in Medicine: Hematology and Oncology (Escola Paulista de Medicina/UNIFESP) with postdoctoral, doctoral, and master's students; and a group of the same name registered with Conselho Nacional de Desenvolvimento Científico e Tecnológico (or CNPq) (Brazil).
**d) Medical assistance**: as the main action in assistance, we began implementing the PBM at the Hospital São Paulo (University Hospital of UNIFESP) in 2019. Through a multidisciplinary group, several actions for implementing PBM were developed, well schematized in flow charts, and simple to understand and execute, according to our implementation model published in this journal^[13]^. Protocols were prepared for the pharmacological treatment of anemia (for 1^st^ pillar) and the management of bleeding (for 2^nd^ pillar) in a practical and easy-to-consult model, in addition to creating an Anemia Ambulatory - PBM. We will soon publish data on improving clinical-surgical and economic outcomes directly related to PBM strategies.

We are supported by international leaders, researchers, and partnerships with various national institutions, including the Sociedade Brasileira de Cirurgia Cardiovascular (or SBCCV) and the Sociedade Brasileira de Neurocirurgia (or SBN), for which we are greatly appreciated.
